# Pathophysiological and prognostic relevance of exercise CMR-derived pulmonary artery compliance in patients with suspected diastolic dysfunction and normal right ventricular function

**DOI:** 10.1093/ehjimp/qyaf077

**Published:** 2025-06-04

**Authors:** Alexander Schulz, Lara Kuttenkeuler, Sören J Backhaus, Torben Lange, Jonas Otto, Judith Gronwald, Ruben Evertz, Johannes T Kowallick, Gerd Hasenfuß, Andreas Schuster

**Affiliations:** Department of Cardiology and Pneumology, University Medical Center Göttingen, Georg-August University, Robert-Koch-Str. 40, Göttingen 37075, Germany; Department of Medicine, Cardiovascular Division, Beth Israel Deaconess Medical Center and Harvard Medical School, 330 Brookline Ave, Boston, MA 02215, USA; German Center for Cardiovascular Research (DZHK), Partner Site Lower Saxony, Niedersachsen, Germany; Department of Cardiology and Pneumology, University Medical Center Göttingen, Georg-August University, Robert-Koch-Str. 40, Göttingen 37075, Germany; German Center for Cardiovascular Research (DZHK), Partner Site Rhine-Main, Bad Nauheim, Germany; Department of Cardiology, Campus Kerckhoff of the Justus-Liebig-University Giessen, Kerckhoff-Clinic, Bad Nauheim, Germany; Department of Cardiology and Angiology, Medical Clinic I, University Hospital Giessen, Justus-Liebig-University Giessen, Giessen, Germany; Department of Cardiology and Pneumology, University Medical Center Göttingen, Georg-August University, Robert-Koch-Str. 40, Göttingen 37075, Germany; German Center for Cardiovascular Research (DZHK), Partner Site Lower Saxony, Niedersachsen, Germany; Fakultät für Mathematik und Informatik, Georg-August Universität Göttingen, Bunsenstraße 3-5, Göttingen 37073, Germany; Department of Cardiology and Pneumology, University Medical Center Göttingen, Georg-August University, Robert-Koch-Str. 40, Göttingen 37075, Germany; German Center for Cardiovascular Research (DZHK), Partner Site Lower Saxony, Niedersachsen, Germany; Department of Cardiology and Pneumology, University Medical Center Göttingen, Georg-August University, Robert-Koch-Str. 40, Göttingen 37075, Germany; German Center for Cardiovascular Research (DZHK), Partner Site Lower Saxony, Niedersachsen, Germany; FORUM Radiology, Rosdorf, Germany; Department of Cardiology and Pneumology, University Medical Center Göttingen, Georg-August University, Robert-Koch-Str. 40, Göttingen 37075, Germany; German Center for Cardiovascular Research (DZHK), Partner Site Lower Saxony, Niedersachsen, Germany; Department of Cardiology and Pneumology, University Medical Center Göttingen, Georg-August University, Robert-Koch-Str. 40, Göttingen 37075, Germany; FORUM Cardiology, Rosdorf, Germany; School of Biomedical Engineering and Imaging Sciences, King’s College London, London, UK

**Keywords:** diastolic dysfunction, heart failure with preserved ejection fraction, cardiovascular magnetic resonance imaging, pulmonary artery compliance, right ventricular dysfunction

## Abstract

**Aims:**

Right ventricular (RV) dysfunction has been associated with worse prognosis in patients with diastolic dysfunction, highlighting the importance of early detection. Main pulmonary artery (MPA) compliance may indicate adverse biventricular coupling prior to emerging RV function.

**Methods and results:**

Sixty-eight patients with suspected diastolic dysfunction (New York Heart Association ≥ II, LV EF ≥ 50%, E/e′ ≥ 8) were prospectively recruited and underwent rest and stress right heart catheterization, echocardiography, and cardiovascular magnetic resonance imaging (CMR) within 24 h. Maximum (*A*_max_) and minimum (*A*_min_) MPA vessel area and stroke volume (RVSV) were obtained from CMR real-time phase-contrast images at rest and during exercise stress. Compliance was calculated as pulsatility $\mathrm{MP}A_{\mathrm{Puls}}=\left(\frac{A_{\max}-A_{\min}}{A_{\min}}\right)\times 100$ and capacitance $\mathrm{MP}A_{\mathrm{Cap}}=\frac{\mathrm{MP}A_{\mathrm{Puls}}}{\mathrm{RVSV}}$. Patients had systematic follow-up after 48 months. Occurrence of cardiovascular events was defined as the primary endpoint. A total of 63 patients [66 ± 9 years, 39 (61.9%) female] were eligible for final analyses. MPA_Puls_ and MPA_Cap_ were lower during exercise stress compared with rest (20% vs. 17.5%, *P* = 0.034 and 0.26%/mL vs. 0.20%/mL, *P* = 0.001). Subgroups with and without heart failure with preserved ejection fraction (HFpEF) had similar MPA compliance at rest, however, HFpEF patients had a steeper decrease of compliance during exercise stress (MPA_Puls_ 13% vs. 20%; *P* < 0.001 MPA_Cap_ 0.16%/mL vs. 0.25%/mL, *P* = 0.018). Decreasing MPA_Puls_ and MPA_Cap_ during exercise stress correlated with markers of diastolic dysfunction including pulmonary capillary wedge pressure, E/e′, and HFA-PEFF score. Patients with decreased MPA_Puls_ (HR 6.0; *P* = 0.016) and MPA_Cap_ (HR 11.3; *P* = 0.015) had worse outcomes independent from conventional markers of diastolic dysfunction.

**Conclusion:**

In patients with suspected diastolic dysfunction and preserved RV function, exercise-stress testing unmasked decreasing CMR-derived MPA compliance, associated with LV diastolic dysfunction and indicated higher risk for cardiovascular events.

## Graphical Abstract

**Graphical Abstract:**
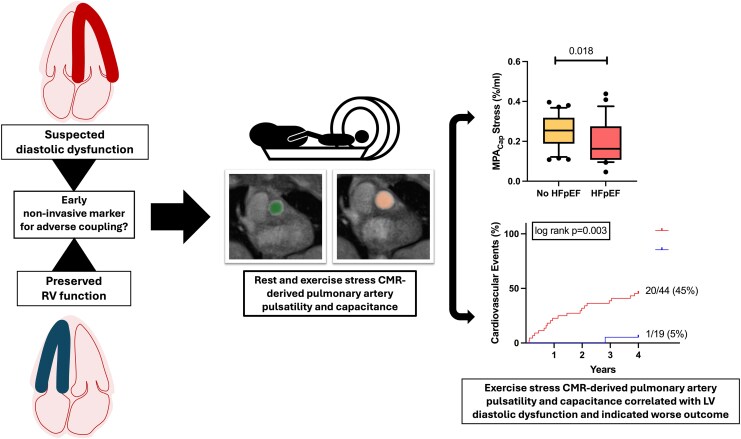
RV, right ventricle; LV, left ventricle; MPA, main pulmonary artery; HFpEF, heart failure with preserved ejection fraction; CMR, cardiovascular magnetic resonance imaging.

## Introduction

Diastolic dysfunction and heart failure with preserved ejection fraction (HFpEF) are now the most common diagnoses among patients with heart failure.^1,2^ Despite significant progress in early diagnosis through clinical scores^3,4^ and advanced imaging modalities,^5^ therapeutic options remain limited, primarily achieving symptom alleviation or reduced hospitalization at best.^6,7^

While some interventions target systemic conditions like obesity^8^ and diabetes^9^ to mitigate heart failure manifestations, therapies that directly address the pathomechanisms of diastolic dysfunction and HFpEF are scarce.

Earlier studies on the interatrial shunt device (IASD)^7^ and ongoing trials investigating the utility of Mavacamten^10^ have primarily focused on left ventricular (LV) pathology, often neglecting the role of the right ventricle (RV). This oversight partly explains the lack of superiority of the IASD compared with sham procedures.^7^ Recognizing this limitation, subsequent investigations from the RESPONDER-HF Trial now incorporate right ventricular and pulmonary vascular remodelling indices for therapy allocation to improve outcomes.^11^

The emerging evidence highlights the prognostic impact of RV dysfunction in patients with diastolic dysfunction, indicating advanced disease stages and explaining worse therapy responses.^12–14^ RV dysfunction results from complex biventricular interactions in diastolic dysfunction, underscoring the need for an improved understanding of biventricular coupling, particularly in patients without manifest RV dysfunction, to prevent progression to advanced stages. In this context, pulmonary artery compliance may serve as a critical gatekeeper in the development of RV dysfunction.^13^

Cardiovascular magnetic resonance imaging (CMR) is a well-established tool for detecting subtle, early changes in myocardial structure and function, allowing for the visualization of pathophysiological mechanisms, diagnosis, and prognostication in diastolic dysfunction and HFpEF.^5,15,16^ Additionally, CMR provides non-invasive surrogates of pulmonary artery compliance, such as pulsatility and capacitance, by leveraging phase-contrast flow imaging.^17–19^

However, in early stages of diastolic dysfunction and HFpEF, as well as in patients without RV dysfunction, subtle changes of impaired compliance may remain undetectable at rest. In patients with diastolic dysfunction, the addition of physiological exercise impacts LV loading conditions and reveals pathological increases in filling pressures^20^ that may, in turn, elicit measurable changes in MPA compliance.

Therefore, we aim to describe the pathophysiological relationship between CMR-based measures of pulmonary artery compliance at rest and during exercise stress in patients with suspected diastolic dysfunction and preserved RV function. The understanding of early adverse biventricular coupling may help to identify patients at higher risk for disease progression and cardiovascular events.

## Methods

### Patient cohort

During the HFpEF stress trial,^5^ 68 symptomatic patients (New York Heart Association ≥ II) with preserved left ventricular ejection fraction (LV EF)≥ 50% and suspicion of diastolic dysfunction (E/e′ ≥ 8) were prospectively enrolled and completed all assessments between 08/2017 and 09/2019. Exclusion criteria included contraindications for CMR (claustrophobia, non-CMR-conditional devices, active bronchospastic disease, or allergy to gadolinium-based contrast agents), pulmonary causes of dyspnoea (forced expiratory volume in 1 s or vital capacity < 80% of the reference), or cardiac conditions responsible for symptomatic burden (coronary artery disease defined as luminal stenosis ≥ 50%, moderate-to-severe valvular heart disease, or the presence of cardiomyopathy).

Eligible patients underwent rest and stress right heart catheterization (RHC) with simultaneous rest and stress transthoracic echocardiography (TTE), followed by rest and exercise stress CMR within 24 h of RHC. Patients had to be in stable sinus rhythm during all assessments.

Patients were diagnosed with HFpEF based on established RHC thresholds: pulmonary capillary wedge pressure (PCWP) ≥ 15 mmHg at rest or ≥25 mmHg during exercise stress.

Normal RV function was defined using CMR-derived RV EF measurements, with thresholds of ≥46% for men and ≥42% for women in the short-axis view, according to guideline recommendations.^21^

All patients underwent systematic follow-up via telephone contact and hospital chart reviews over 48 months, completed between 08/2023 and 10/2023. The clinical endpoint was the occurrence of cardiovascular events.

The study was approved by the local ethics committee at the University of Göttingen. Written informed consent was obtained from all participants before enrolment. The study adhered to the principles of the Helsinki Declaration and was funded by the German Centre for Cardiovascular Research (DZHK-17).

### RHC with simultaneous echocardiography

Rest and exercise stress RHC was conducted using an established ramp protocol on a supine ergometer, with incremental increases of 5 W to achieve and maintain a target heart rate of 100–110 bpm. Haemodynamic measurements included mean pulmonary artery pressure (mPAP), PCWP, and pulmonary vascular resistance (PVR).

Simultaneously, rest and stress TTE assessments were performed to evaluate biventricular and left atrial morphology and function. Additionally, diastolic function was assessed using pulsed-wave, continuous-wave, and tissue Doppler imaging to obtain detailed measurements.

### Exercise stress CMR

CMR was performed using a 3.0T Magnetom Skyra MRI scanner (Siemens Healthcare, Erlangen, Germany) equipped with a 32-channel cardiac surface receiver coil. The same exercise ramp protocol as used during RHC was applied, utilizing a dedicated CMR-compatible ergometer (Lode, Leiden, Netherlands).

Resting biventricular volumes and function were assessed using balanced steady-state free precession (bSSFP) cine sequences, including long-axis views (four-, three-, and two-chamber) and a short-axis stack with full ventricular and atrial coverage [25 frames per cardiac cycle, time of echo (TE) 1.5 ms, time of repetition (TR) 55 ms, flip angle 55°, slice thickness 7 mm, inter-slice gap 7.7 mm]. Global longitudinal strain measurements were derived from the average of three consecutive assessments in long-axis orientation using commercially available software (2D CPA MR, Cardiac Performance Analysis, TomTec Imaging Systems, Unterschleißheim, Germany).^22^

**Figure 1:**
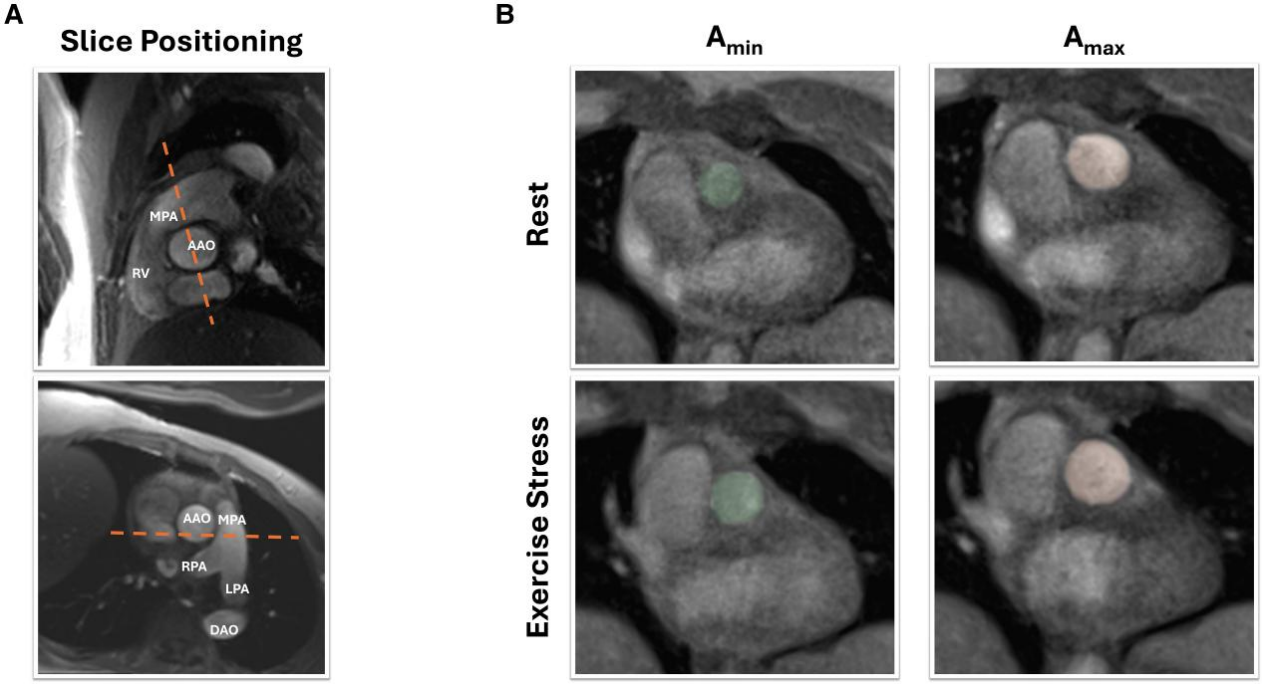
(*A*) Slice positioning to derive MPA flow measurements. The dashed line indicates the slice position to obtain real-time phase-contrast flow measurements of the main pulmonary artery. (*B*) Examples of the minimum (*A*_min_) and maximum (*A*_max_) area of the main pulmonary artery at rest and during exercise stress to subsequently calculate MPA_Puls_ and MPA_Cap_. A, area; MPA_Puls_, main pulmonary artery pulsatility; MPA_Cap_, main pulmonary artery capacitance; AAO, ascending aorta; DAO, descending aorta; RV, right ventricle; MPA, main pulmonary artery; RPA, right pulmonary artery; LPA, left pulmonary artery.

Real-time phase-contrast flow imaging (temporal resolution 44 ms, minimum TE/TR of 3.15/2.46 ms) was performed using established sequences at rest and during exercise stress.^23^ As shown in *Figure 1A*, measurements were obtained in the pulmonary artery distal to the pulmonary valve but proximal to its bifurcation into the left and right pulmonary arteries.

Diffuse myocardial fibrosis was quantified using T1 mapping in a single mid-ventricular short-axis slice with a Modified Look-Locker Inversion Recovery (MOLLI) technique (FOV of 360 × 306.6 mm^2^, in-plane resolution 1.41 × 1.41 × 8 mm^3^, TR 280 ms, TE 1.12 ms, TI 180 ms, flip angle 35°, bandwidth 1085 Hz/pixel with total acquisition of 11 heart beats), both pre- and post-contrast administration. Extracellular volume (ECV) was calculated from the T1 maps as previously described.^24^

### Pulmonary artery compliance

Measurements of MPA compliance were derived from real-time phase-contrast flow acquisitions using cvi42 (version 5.13, Circle Cardiovascular Imaging, Calgary, Canada).

All acquired series were systematically screened for image quality and artefact presence prior to measurements.

Non-invasive measurements of compliance included MPA pulsatility (MPA_Puls_) and MPA capacitance (MPA_Cap_). MPA_Puls_ was derived as the mean relative area change of the MPA at rest and during exercise stress over five consecutive cardiac cycles. If premature ventricular excitations occurred, the referring cardiac cycle and the following cycle were excluded from calculations. As displayed in *Figure 1B*, pulmonary artery borders and area (*A*) were manually delineated at end-diastole (*A*_min_) and end-systole (*A*_max_) and MPA_Puls_ was calculated as $\left(\frac{A_{\max}-A_{\min}}{A_{\min}}\right)\times 100$. For calculation of MPA_Cap_, RV stroke volume (RVSV) was derived from the same cardiac cycles as the MPA area and subsequently calculated as the relative increase of vessel area per millilitre of RVSV: $\mathrm{MP}A_{\mathrm{Cap}}=\frac{\mathrm{MP}A_{\mathrm{Puls}}}{\mathrm{RVSV}}$.^17^ Measurements required for MPA_Puls_ and MPA_Cap_ were taken blinded to other results of RHC, CMR, and TTE. Inter- and intra-observer variability was assessed in 10 randomly selected cases three months after and blinded to initial measurements.

### Statistical analyses

Statistical analyses were performed using SPSS version 29.0.2.0 (IBM, Armonk, NY, USA) and GraphPad Prism 10 (GraphPad Software, San Diego, CA, USA). The Shapiro–Wilk test was used to assess normality of data distribution. Categorical variables are presented as frequencies and percentages and were compared using the χ^2^ test. Continuous variables are reported as medians with inter-quartile ranges (IQRs) and were analysed using the nonparametric Mann–Whitney *U* test or Wilcoxon rank test, as appropriate. Measurements of MPA compliance were correlated with markers of right and left ventricular function using Spearman’s rank correlation, with correlation coefficients reported. Outcome analyses were conducted using log-rank comparisons and visualized with Kaplan–Meier plots. The association of MPA compliance parameters with outcome events was assessed using uni- and multivariable Cox-regression analyses. Models included conventional indicators of diastolic dysfunction, adjusted for age, sex, and body mass index (BMI). Variables showing significant associations in univariable analyses were included in the multivariable models. Inter- and intra-observer variability was evaluated using intra-class correlation coefficients (ICC) with corresponding 95% confidence intervals (CI). A *P*-value of <0.05 was considered statistically significant.

## Results

### Patient cohort and repeatability of measurements

Of a total of 68 patients, five patients had to be excluded due to significant motion corruption of the acquisition (*n* = 4) or high burden of premature ventricualr excitations during phase-contrast imaging of the MPA (*n* = 1). The final study population consisted of 63 patients (67 ± 9 years, 38% male). *Table 1* shows characteristics of the overall cohort. All patients were found to have preserved RV function (RV EF 65.5%, range: 50.0–76.9%).

**Table 1:** Baseline characteristics of the cohort including echocardiography, right heart catheterization, and cardiovascular magnetic resonance imaging

Baseline characteristics	
Age (years)	70 (66; 75)
BMI (kg/m^2^)	28.1 (26.1; 32.4)
NT-proBNP (ng/L)	133.5 (67.9; 268.1)
HFpEF (*n*, %)	30 (48)
Male sex (*n*, %)	24 (38)
NYHA II/III (*n*, %)	44/19 (70/30)
Diabetes (*n*, %)	9 (14)
AHT (*n*, %)	51 (81)
AFIB (*n*, %)	20 (32)
DLP (*n*, %)	39 (62)
Smoking (*n*, %)	9 (14)
HFA-PEFF score	4 (3; 6)
Echocardiography	
E/e′ rest	10.7 (8.7; 12.8)
E/e′ stress	12.1 (10.5; 15.2)
LAVI (mL/m^2^)	44.3 (36.2; 56.2)
TAPSE (mm)	23.7 (21.2; 26.5)
PAPsys (mmHg)	24.0 (21.0; 32.3)
Right heart catheterization	
PCWP rest (mmHg)	11.0 (8.0; 17.0)
PCWP stress (mmHg)	25.0 (19.0; 28.0)
mPAP rest (mmHg)	20.0 (16.0; 25.0)
mPAP stress (mmHg)	40.0 (34.0; 47.0)
PVR rest (Wood)	1.7 (1.1; 2.1)
PVR stress (Wood)	2.5 (1.2; 2.4)
Cardiovascular magnetic resonance imaging	
LVMI (g/m^2^)	58.1 (50.9; 68.7)
LVEDVi (mL/m^2^)	69.7 (59.4; 77.8)
LVESVi (mL/m^2^)	20.5 (15.0; 26.0
LV EF (%)	69.0 (66.0; 69.5)
LV GLS (%)	−20.6 (−22.9; −18.9)
RVEDVi (mL/m^2^)	67.2 (58.4; 74.0)
RVESVi (mL/m^2^)	21.0 (18.6; 27.9)
RV EF (%)	65.5 (61.0; 69.5)
RV GLS (%)	−22.9 (−26.5; −20.2)

Values are provided in frequencies with corresponding percentages for categorical variables or as a median with corresponding inter-quartile range for continuous variables.

BMI, body mass index; HFpEF, heart failure with preserved ejection fraction; NYHA, New York Heart Association; AHT, arterial hypertension; AFIB, atrial fibrillation; DLP, dyslipoproteinaemia; TAPSE, tricuspid annular plane systolic excursion; E, passive mitral inflow; e′, septal and lateral mitral annulus velocity; PCWP, pulmonary capillary wedge pressure; mPAP, mean pulmonary artery pressure; PVR, pulmonary vascular resistance; PAPsys, pulmonary artery systolic pressure; LVMI, left ventricular mass index; LAVI, left atrial volume index; GLS, global longitudinal strain; NT-proBNP, N-terminal prohormone of brain natriuretic peptide; EDVi/ESVi, end-diastolic/-systolic volume index; EF, ejection fraction; LV/RV, left/right ventricular.

Assessments of MPA_Puls_ had excellent intra- and inter-observer variability at rest [intra-observer ICC: 0.98 (95% CI: 0.93–0.99; *P* < 0.001); inter-observer ICC: 0.90 (95% CI: 0.60–0.98; *P* = 0.001)] and during exercise stress [intra-observer ICC: 0.97 (95% CI: 0.91–0.99; *P* < 0.001); inter-observer ICC: 0.94 (95% CI: 0.77–0.99; *P* < 0.001)].

### Non-invasive assessments of main pulmonary artery compliance at rest and during exercise stress

**Figure 2:**
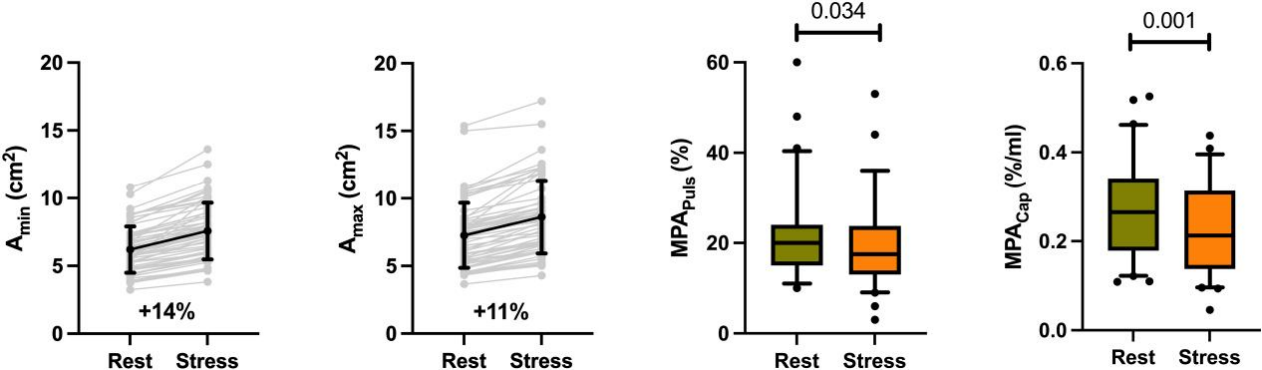
Measurements of MPA area, cardiovascular magnetic resonance-derived MPA_Puls_ and MPA_Cap_ at rest and during exercise stress. Given percentages indicate relative change in area from rest to stress. A, area; MPA_Puls_, main pulmonary artery pulsatility; MPA_Cap_, main pulmonary artery capacitance.

*Figure 2* details measurements of MPA_Puls_ and MPA_Cap_ at rest and during exercise. Both measurements indicated lower MPA compliance during exercise stress compared with resting conditions [MPA_Puls_: 20.0% (IQR 15.0–24.0%) vs. 17.5% (IQR: 13.0–23.8%), *P* = 0.034; MPA_Cap_: 0.26%/mL (IQR 0.18–0.36) vs. 0.20%/mL (IQR 0.14–0.31); *P* = 0.001].

While both, *A*_min_ and *A*_max_ significantly increased from rest to stress (*A*_min_: 5.8 cm^2^ vs. 7.1 cm^2^; *P* < 0.001; *A*_max_: 7.3 cm^2^ vs. 8.3 cm^2^; *P* < 0.001), relative increase was steeper for *A*_min_ compared with *A*_max_ [+14% (5; 26) vs. +11% (5; 23); *P* = 0.041].

Compared with female patients, male patients demonstrated similar MPA_Puls_ at rest [21.0% (IQR 15.5–25.0) vs. 19.0% (IQR 14.8–23.0), *P* = 0.391] and during exercise [19.0% (IQR 13.0–23.5) vs. 16.5% (IQR 12.8–25.8), *P* = 0.902]. In contrast, MPA_Cap_ was significantly higher in female patients both at rest [0.30%/mL (IQR 0.22–0.40) vs. 0.18%/mL (IQR 0.13–0.26), *P* < 0.001] and during exercise stress [0.25%/mL (IQR 0.17–0.34) vs. 0.16%/mL (IQR 0.11–0.25), *P* = 0.039]. No significant differences in MPA_Puls_ or MPA_Cap_ at rest or during exercise were observed between patients with and without atherosclerotic risk factors such as diabetes mellitus (*P* > 0.140), dyslipoproteinaemia (*P* > 0.379), arterial hypertension (*P* > 0.363), or obesity (*P* > 0.506).

### Differences of main pulmonary artery compliance at rest and during exercise stress in patients with and without HFpEF

**Figure 3:**
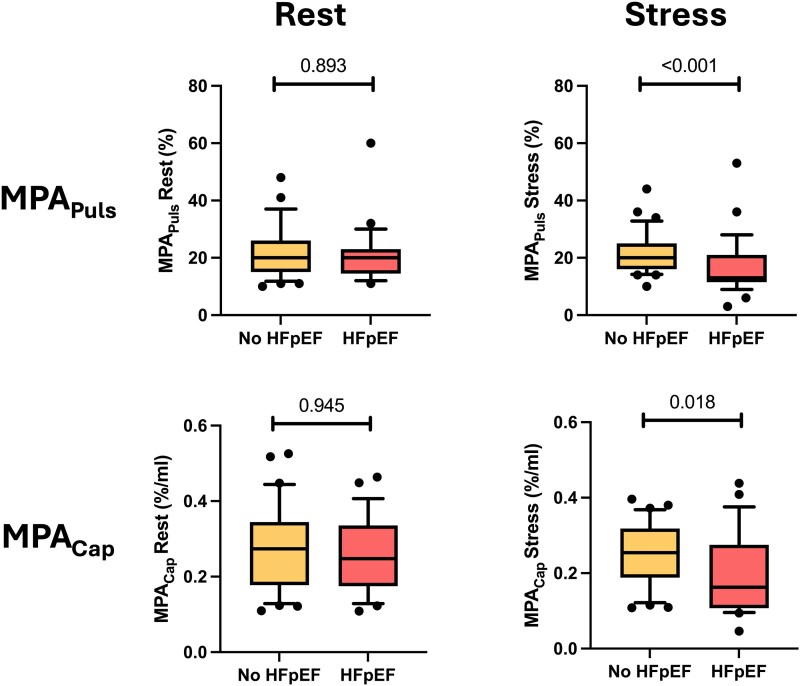
Measurements of cardiovascular magnetic resonance-derived MPA_Puls_ and MPA_Cap_ at rest and during exercise stress in patients with and without HFpEF. HFpEF, heart failure with preserved ejection fraction; MPA_Puls_, main pulmonary artery pulsatility; MPA_Cap_, main pulmonary artery capacitance.

*Table 2* shows details on morphological and functional characteristics of non-invasive and invasive measurements in the subgroup of patients with final diagnosis of HFpEF and the remaining patients with suspected diastolic dysfunction. As displayed in *Figure 3*, patients with HFpEF had similar MPA_Puls_ and MPA_Cap_ at rest compared with patients without. However, both measures indicated lower MPA compliance during exercise in HFpEF patients compared with patients without HFpEF [MPA_Puls_ during exercise: 13% (IQR 11.5–21) vs. 20% (IQR 16–25); *P* < 0.001, MPA_Cap_ during exercise: 0.16%/mL (0.11–0.28) vs. 0.25%/mL (0.19–0.32); *P* = 0.018].

**Table 2:** Functional and morphological characteristics of patients with and without HFpEF

Parameter	No HFpEF (*n* = 33)	HFpEF (*n* = 30)	*P*-value
Echocardiography			
E/e′ rest	8.9 (7.5; 9.8)	12.6 (10.0; 13.6)	<0.001
E/e′ stress	11.0 (10.0; 14.0)	13.8 (11.0; 17.0)	0.119
LAVI (mL/m^2^)	37.8 (30.4; 46.1)	50.1 (39.1; 59.0)	<0.001
TAPSE (mm)	22.8 (20.7; 26.4)	23.9 (20.5; 26.9)	0.412
PAPsys (mmHg)	21.3 (18.9; 24.3)	29.5 (23.9; 34.6)	0.001
Right heart catheterization			
PCWP rest (mmHg)	7 (5.0; 10.8)	15 (11.3; 18.8)	<0.001
PCWP stress (mmHg)	19 (12.3; 22.0)	28 (25.3; 31.0)	<0.001
mPAP rest (mmHg)	16 (12.3; 17)	24 (20; 29.8)	<0.001
mPAP stress (mmHg)	34 (26.3; 37.5)	46 (40; 55.3)	<0.001
PVR rest (Wood)	1.6 (0.9; 1.8)	1.7 (1.2; 2.4)	0.143
PVR stress (Wood)	1.3 (1.0; 1.9)	1.7 (1.3; 2.9)	0.116
Cardiovascular magnetic resonance imaging			
LVMI (g/m^2^)	58.1 (50.8; 73.8)	57.0 (50.8; 66.2)	0.912
LVEDVi (mL/m^2^)	74.1 (58.3; 77.3)	68.3 (59.9; 80.2)	0.680
LVESVi (mL/m^2^)	19.8 (14.7; 27.1)	21.5 (14.9; 26.7)	0.587
LV EF (%)	69.3 (65.1; 75.8)	68.0 (65.9; 75.6)	0.901
LV GLS (%)	−21.0 (−23.4; −19.4)	−20.0 (−22.5; −18.8)	0.145
RVEDVi (mL/m^2^)	67.6 (57.1; 85.2)	67.9 (54.6; 71.9)	0.967
RVESVi (mL/m^2^)	25.0 (18.9; 29.8)	20.6 (16.6; 24.6)	0.280
RV EF (%)	64.1 (60.3; 69.4)	67.3 (63.8; 70.9)	0.104
RV GLS (%)	−22.9 (−26.6; −20.2)	−23.1 (−26.7; −20.4)	0.815
T1 (ms)	1195 (1175; 1221)	1203 (1167; 1234)	0.421
ECV (%)	25.0 (23.3; 26.9)	25.5 (23.2; 28.3)	0.578
MPA_Puls_ rest (%)	20 (15; 25)	20 (15; 23.8)	0.893
MPA_Puls_ stress (%)	20 (16; 25)	13 (11.3; 21)	<0.001
MPA_Cap_ rest (%/mL)	0.27 (0.18; 0.35)	0.26 (0.19; 0.36)	0.945
MPA_Cap_ stress (%/mL)	0.25 (0.19; 0.32)	0.16 (0.11; 0.28)	0.018

Values are provided in frequencies with corresponding percentages for categorical variables or as a median with corresponding inter-quartile range for continuous variables.

BMI, body mass index; HFpEF, heart failure with preserved ejection fraction; TAPSE, tricuspid annular plane systolic excursion; E, passive mitral inflow; e′, septal and lateral mitral annulus velocity; PCWP, pulmonary capillary wedge pressure; mPAP, mean pulmonary artery pressure; PVR, pulmonary vascular resistance; PAPsys, pulmonary artery systolic pressure; LVMI, left ventricular mass index; LAVI, left atrial volume index; GLS, global longitudinal strain; NT-proBNP, N-terminal prohormone of brain natriuretic peptide; EDVi/ESVi, end-diastolic/-systolic volume index; EF, ejection fraction; MPA_Puls_, main pulmonary artery pulsatility; MPA_Cap_, main pulmonary artery capacitance; LV/RV, left/right ventricular.

### Correlation of non-invasive main pulmonary artery pulsatility and capacitance with systolic and diastolic functional parameters

*Table 3* displays the correlation of MPA_Puls_ and MPA_Cap_ at rest and during exercise stress with LV and RV systolic function as well as echocardiographic, RHC, and CMR markers of diastolic dysfunction. While there was no correlation of non-invasive MPA compliance with biventricular systolic function, exercise assessments of both parameters revealed moderate negative correlation with measures of diastolic function including the HFA-PEFF score (MPA_Puls_: *r* = −0.455; *P* < 0.001; MPA_Cap_: *r* = −0.421; *P* < 0.001), PCWP at rest (MPA_Puls_: *r* = −0.444; *P* < 0.001, MPA_Cap_: *r* = −0.371; *P* = 0.004), and PCWP during exercise stress (MPA_Puls_: *r* = −0.492;*P* < 0.001, MPA_Cap_:-0.396; *P* = 0.002). Decreasing MPA_Puls_ during exercise was also correlated with increasing E/e′ at rest (*r* = −0.361; *P* = 0.005) and during exercise stress (*r* = −0.325; *P* = 0.032).

**Table 3:** Correlation of main pulmonary artery pulsatility and capacitance with markers of systolic and diastolic cardiac function and pulmonary vascular remodelling

*Parameter*	MPA_Puls_	*P*-value	MPA_Puls_ Stress	*P*-value	MPA_Cap_	*P*-value	MPA_Cap_	*P*-value
	Rest				Rest		Stress	
*LV EF*	0.032	0.806	0.210	0.107	0.111	0.386	0.179	0.175
*RV EF*	0.129	0.313	0.010	0.941	0.20	0.070	0.186	0.159
*LV GLS*	−0.201	0.114	−0.218	0.095	−0.089	0.489	−0.014	0.916
*RV GLS*	0.125	0.330	0.018	0.893	−0.100	0.433	−0.101	0.446
*HFA-PEFF*	0.031	0.812	**−0**.**455**	**<0**.**001**	0.017	0.897	**−0**.**421**	**<0**.**001**
*E/e rest*	−0.008	0.950	**−0**.**361**	**0**.**005**	0.248	0.050	−0.142	0.283
*E/e stress*	−0.178	0.231	**−0**.**325**	**0**.**032**	0.218	0.141	−0.025	0.872
*PCWP* r*est*	0.091	0.480	**−0**.**444**	**<0**.**001**	−0.052	0.−687	**−0**.**371**	**0**.**004**
*PCWP* s*tress*	−0.100	0.435	**−0**.**492**	**<0**.**001**	−0.121	0.346	**−0**.**396**	**0**.**002**

Bold values indicate statistical significance. E, passive mitral inflow; e′, septal and lateral mitral annulus velocity; PCWP, pulmonary capillary wedge pressure; GLS, global longitudinal strain; LV/RV, left/right ventricular; EF, ejection fraction; MPA_Puls_, main pulmonary artery pulsatility; MPA_Cap_, main pulmonary artery capacitance.

### Risk stratification using non-invasive measurements of main pulmonary artery compliance

One patient was lost to follow-up. A total of 21 (33%) patients reached the primary endpoint after a mean follow-up period of four years. Using Youden’s index, an optimal cut-off for prediction of the primary endpoint using MPA_Puls_ was found at 22.5% and using MPA_Cap_ at 0.31%/mL. Supplementary data online, *Tables S1* and *S2* show the characteristics of the subgroups with low and high MPA_Puls_ and MPA_Cap_, respectively. Patients with low MPA_Puls_ and MPA_Cap_ had a history of atrial fibrillation more frequently and more prominent diastolic dysfunction as appreciated by echocardiography and RHC without any differences in other baseline characteristics or CMR measurements including diffuse myocardial fibrosis.

**Figure 4:**
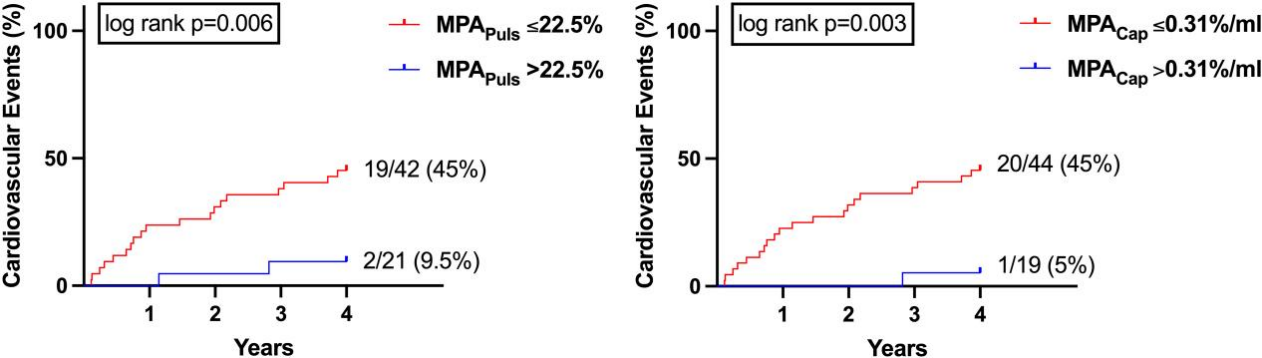
Kaplan–Meier plots indicating cardiovascular events in patients with low and high MPA_Puls_ and MPA_Cap_ during exercise stress. MPA_Puls_, main pulmonary artery pulsatility; MPA_Cap_, main pulmonary artery capacitance.

As shown in *Figure 4*, both parameters enabled risk stratification of cardiovascular events, as patients with lower MPA_Puls_ and MPA_Cap_ had a significantly higher rate of cardiovascular events compared with patients with higher measures of MPA compliance (MPA_Puls_ log-rank *P* = 0.003, MPA_Cap_ log-rank *P* = 0.003). In univariable regression analyses, both parameters were associated with the primary endpoint [MPA_Puls_: HR 6.0 (95% CI: 1.4–25.9), *P* = 0.016, MPA_Cap_: HR 11.3 (95% CI: 1.5–84.2), *P* = 0.015]. As seen in *Table 4*, both reduced MPA_Puls_ and reduced MPA_Cap_ remained independently associated with the primary endpoint in a multivariable regression model incorporating conventional parameters characterizing diastolic dysfunction [MPA_Puls_: 5.1 (1.1–24.1); *P* = 0.039, MPA_Cap_: 9.6 (1.2–75.8); *P* = 0.032].

**Table 4:** Uni- and multivariable Cox-regression analyses

Parameter	Univariable HR	*P*-value	Multivariable HR^a^	*P*-value
Model 1: main pulmonary artery pulsatility				
MPA_Puls_	6.0 (1.4–25.9)	0.016	5.1 (1.1–24.1)	0.039
HFA-PEFF score	1.3 (1.0–1.8)	0.070		
NT-proBNP	1.1 (1.1–1.1)	<0.001	1.0 (1.0–1.0)	0.170
E/e′	1.1 (1.0–1.1)	0.030	0.9 (0.8–1.0)	0.168
PCWP	1.1 (1.0–1.1)	0.010	1.0 (0.9–1.1)	0.726
LAVI	1.1 (1.0–1.1)	<0.001	1.0 (1.0–1.1)	0.097
Model 2: main pulmonary artery capacitance				
MPA_Cap_	11.3 (1.5–84.2)	0.018	9.6 (1.2–75.8)	0.032
HFA-PEFF score	1.3 (1.0–1.8)	0.070		
NT-proBNP	1.1 (1.1–1.1)	<0.001	1.0 (1.0–1.0)	0.086
E/e′	1.1 (1.0–1.1)	0.030	0.9 (0.8–1.1)	0.233
PCWP	1.1 (1.0–1.1)	0.010	1.0 (0.9–1.1)	0.712
LAVI	1.1 (1.0–1.1)	<0.001	1.0 (1.0–1.1)	0.224

HR, hazard ratio; E, passive mitral inflow; e′, septal and lateral mitral annulus velocity; PCWP, pulmonary capillary wedge pressure; LAVI, left atrial volume index; NT-proBNP, N-terminal prohormone of brain natriuretic peptide; MPA_Puls_, main pulmonary artery pulsatility; MPA_Cap_, main pulmonary artery capacitance.

^a^Adjusted for age, sex, and BMI.

## Discussion

This study demonstrates that MPA_Puls_ and MPA_Cap_, as non-invasive measures of MPA compliance, effectively detect prognostically relevant changes in biventricular coupling during exercise stress in patients with suspected diastolic dysfunction and preserved RV systolic function. These findings highlight the importance of biventricular assessments in diastolic dysfunction and emphasize the value of non-invasive methods for identifying adverse haemodynamic conditions in these patients.

RV dysfunction is a well-established predictor of adverse outcomes in patients with diastolic dysfunction^25,26^ and diminishes efficacy of therapeutic approaches.^11^ As a result, early detection of alterations in biventricular loading and coupling is critical before overt RV dysfunction develops.

In patients with diastolic dysfunction, RV impairment is driven by increased transpulmonary gradients and adverse RV afterloading, which may initially manifest as early changes in MPA compliance. If this persistent loading remains unrelieved, it can result in elevated RV strain and, over time, lead to progressive RV dysfunction.^27^

This intricate biventricular interrelationship aligns with our findings, demonstrating significant correlations between parameters of diastolic dysfunction and measures of MPA compliance. While previous studies have reported diminished MPA compliance in heart failure populations already; these cohorts predominantly included patients at more advanced disease stages with often established RV dysfunction,^13,27^ or assessments conducted after acute HF decompensation.^28^ This study expands on these findings by demonstrating that patients with preserved RV function may also encounter changes in MPA compliance with relevance to their prognosis. Crucially, these alterations in MPA compliance were detectable during exercise stress only and remained concealed at rest.

Despite the fact that changes in MPA compliance were more pronounced in the subgroup of patients with HFpEF, significant changes were already observed in the overall cohort having unclear dyspnoea and suspected diastolic dysfunction. This highlights the value of stress testing not only for LV function but also for detection of adverse biventricular coupling and risk prediction in early disease stages.

Pathophysiologically, intact MPA compliance may act as a buffer for increased LV afterload in patients with early-stage diastolic dysfunction,^29^ allowing compensation for elevated afterload under resting conditions. However, during exercise, limited compliance reserve, i.e. inadequate increase of vessel diameter, may result in greater pressure loading transmitted to the RV, ultimately impairing its function. This aligns with our findings of a steeper increase in minimal vessel area compared with maximal vessel area during exercise stress, suggesting that afterloading disproportionately affects the vessel’s baseline dimensions. While the minimal area, which reflects the vessel’s ground load, is incrementally expanded, maximum area cannot expand unlimited causing a reduced compliance reserve during exercise. However, this hypothesis warrants further pre-clinical investigation.

In addition, we observed sex-specific differences in MPA_Cap_, with women exhibiting higher values than men. This finding may reflect intrinsic differences in vascular responsiveness between sexes and may align with previous reports of sex-specific variation in biventricular coupling and functional reserve.^30^ Future studies exploring haemodynamic responses in patients with diastolic dysfunction should consider including measures of vascular compliance to deepen our understanding of underlying pathophysiological mechanisms.

Although vascular compliance can be assessed using invasive methods,^12^ non-invasive approaches are highly desirable for early and continuous risk stratification, as well as for individualized diagnostic strategies that are more practical for routine clinical use. While echocardiographic methods have been proposed,^28^ they face challenges such as limited visualization of right heart structures and declining image quality during exercise. In contrast, CMR-based assessments offer free angulation, high repeatability, and user-friendly measurements, potentially making them promising parameters for a more frequent application and prospective validation.

Even though this study should be regarded as a proof-of-concept for the derivation and clinical application of CMR-derived measures of MPA compliance, the proposed parameters may hold important clinical implications. First, they offer the potential to detect early reductions in MPA compliance during exercise, which may indicate an increased risk of adverse outcomes even in the early stages of disease. This makes them particularly valuable for newly diagnosed patients with preserved biventricular function, supporting risk stratification and guiding decisions on closer monitoring or early therapeutic intervention. Moreover, exercise-based assessment of MPA compliance could be extended to individuals at risk for diastolic dysfunction—even prior to a definitive diagnosis of HFpEF—as our findings revealed prognostically relevant alterations in patients with suspected but not confirmed HFpEF. This may provide novel insights into pre-clinical stages of adverse pathophysiological remodelling. While the complexity of exercise stress CMR currently limits its use for routine screening or broad application, a deeper understanding of impaired biventricular coupling and vascular mechanics may facilitate more individualized therapeutic strategies, particularly for patients who have not yet developed overt RV dysfunction. Finally, the early involvement of MPA compliance in the context of diastolic dysfunction may represent a promising target for novel therapeutic approaches or preventive strategies in at-risk populations.

## Limitations

This study was conducted as a single-centre investigation at a highly experienced facility using advanced CMR imaging techniques. As such, the results may not be generalizable to other centres. Even though RHC and CMR have been performed within 24 h without changes in medication, small changes in haemodynamical conditions cannot be fully excluded. Additionally, the relatively small sample size may limit the generalizability of the findings, and results could differ in a larger validation cohort. Small changes in slice position during exercise may have impacted the results but are expected to be minimized by averaging multiple beats. This *in vivo* study was unable to determine whether actual changes in the vessel wall, potentially reflecting histological correlates of reduced compliance, were present. The limited temporal and spatial resolution of real-time imaging may have influenced the accuracy of the measurements, and patient motion could have introduced further variability. Despite these limitations, the repeatability of the assessments was high, and averaging measurements over five cardiac cycles likely improved accuracy.

## Conclusion

In patients with suspected diastolic dysfunction and preserved RV function, exercise-stress assessments revealed decreasing MPA compliance as non-invasively measured by CMR. Diminished MPA compliance during exercise stress was directly correlated with markers of diastolic dysfunction and indicated a higher probability for cardiovascular events in affected patients. By identifying subtle changes of MPA compliance, these findings highlight the value of stress testing for early risk stratification and individualized interventions to prevent progression and RV remodelling in patients with early diastolic dysfunction and HFpEF.

## Supplementary Material

qyaf077_Supplementary_Data

## Data Availability

Data generated or analysed during the study are available from the corresponding author by request.

## Lead author biography

Dr Alexander Schulz is a cardiology fellow at the University Medical Center Göttingen, Germany, and a Postdoctoral Research Fellow at Beth Israel Deaconess Medical Center and Harvard Medical School, USA. He specializes in multimodality cardiovascular imaging, with a particular focus on cardiovascular magnetic resonance imaging and is a certified Senior Instructor in Cardiovascular Magnetic Resonance Imaging and Cardiovascular Computed Tomography. His research focuses on non-invasive imaging in heart failure with preserved ejection fraction and exercise stress cardiovascular magnetic resonance imaging.
