# Supplementary material for: Pathophysiological and prognostic relevance of exercise CMR-derived pulmonary artery compliance in patients with suspected diastolic dysfunction and normal right ventricular function
Source: Eur Heart J Imaging Methods Pract. 2025 Jun 4;3(1):qyaf077. doi: 10.1093/ehjimp/qyaf077 (PMC12204200; doi:10.1093/ehjimp/qyaf077)
Supplement: qyaf077_Supplementary_Data [file qyaf077_supplementary_data.docx]

**Supplemental Data**

Supplemental Table 1: Characteristics of patients with low and high main pulmonary artery pulsatility

| Parameter | MPA_Puls_ ≤22.5% (n=42) | MPA_Puls_ >22.5% (n=21) | p-value |
| --- | --- | --- | --- |
| Baseline characteristics | | | |
| Age (years) | 69 (66;75) | 68 (66;70) | 0.133 |
| BMI (kg/m^2^) | 28.1 (25.1;32.4) | 27.5 (26.1;31.3) | 0.339 |
| NT-proBNP (ng/l) | 159.9 (76.5;293.9) | 66.7 (43.3;140.2) | 0.004 |
| HFpEF (n,%) | 24 (57) | 6 (29) | 0.032 |
| Male sex (n,%) | 16 (38) | 8 (38) | 1 |
| NYHA II/III (n,%) | 29/13 (69/31) | 15/6 (71/29) | 0.846 |
| Diabetes (n,%) | 5 (12) | 4 (19) | 0.445 |
| AHT (n,%) | 35 (83) | 16 (76) | 0.496 |
| AFIB (n,%) | 19 (45) | 1 (4.8) | 0.001 |
| DLP (n,%) | 25 (60) | 14 (67) | 0.582 |
| Smoking (n,%) | 6 (14) | 3 (14) | 1 |
| HFA PEFF Score | 5 (4;6) | 3 (2;5) | 0.006 |
| Echocardiography | | | |
| E/e’ Rest | 11.2 (9.2;13.1) | 9.0 (7.6;9.9) | 0.021 |
| E/e’ Stress | 14.0 (10.5;16.8) | 11.0 (10.1;12.2) | 0.034 |
| LAVI (ml/m^2^) | 46.8 (37.0;56.7) | 42.6 (29.5;53.6) | 0.094 |
| TAPSE (mm) | 23.3 (21.2;27.0) | 24.0 (19.1;26.5) | 0.835 |
| PAPsys (mmHg) | 27.5 (21.5;33.0) | 23.0 (20.0;24.8) | 0.051 |
| Right heart catheterization | | | |
| PCWP rest (mmHg) | 11.0 (10.0;18.0) | 7.0 (5.0;13.0) | 0.029 |
| PCWP rtress (mmHg) | 26.0 (22.0;30.0) | 18.0 (13.0;22.0) | 0.016 |
| mPAP rest (mmHg) | 21.0(18.0;27.0) | 16.0(14.0;20.0) | 0.062 |
| mPAP rtress (mmHg) | 43.0 (36.0;48.0) | 36.0 (27.0;42.0) | 0.042 |
| PVR rest (Wood) | 1.7 (1.1;2.3) | 1.6 (1.0;1.9) | 0.589 |
| PVR stress (Wood) | 1.5 (1.2;2.4) | 1.4(1.2;1.9) | 0.330 |
| Cardiovascular magnetic resonance imaging | | | |
| LVMI (g/m^2^) | 60.0 (51.8;71.4) | 55.7 (46.2;64.7) | 0.919 |
| LVEDVi (ml/m^2^) | 69.9 (59.1;76.6) | 68.9 (59.4;78.4) | 0.726 |
| LVESVi (ml/m^2^) | 22.2 (16.6;25.8) | 19.1 (14.0;26.8) | 0.287 |
| LVEF (%) | 68.9(66.0;75.9) | 69.3 (64.5;75.7) | 0.307 |
| LV GLS (%) | -20.3 (-22.8;-18.9) | -21.0 (-23.2;-19.0) | 0.382 |
| RVEDVi (ml/m^2^) | 67.9 (56.8;75.9) | 66.4 (58.4;71.6) | 0.521 |
| RVESVi (ml/m^2^) | 22.7 (18.8;27.7) | 19.7 (17.2;28.1) | 0.435 |
| RVEF (%) | 65.9 (62.4;69.3) | 64.6;58.5;70.9) | 0.850 |
| RV GLS (%) | -23.0 (-26.6;-20.3) | -23.0 (-28.4;-20.7) | 0.988 |
| T1 (ms) | 1206 (1186;1235) | 1193 (1168;1235) | 0.297 |
| ECV (%) | 25.9 (24.3;28.1) | 25.5 (23.7;27.0) | 0.180 |

Values are provided in frequencies with corresponding percentages for categorical variables or as a median with corresponding inter-quartile range for continuous variables. BMI – Body mass index, HFpEF – heart failure with preserved ejection fraction, TAPSE – Tricuspid annular plane systolic excursion, E – passive mitral inflow, e’ – septal and lateral mitral annulus velocity, PCWP – pulmonary capillary wedge pressure, mPAP – mean pulmonary artery pressure, , PVR – pulmonary vascular resistance, PAPsys – pulmonary artery systolic pressure, LVMI – left ventricular mass index, LAVI – left atrial volume index, NT-proBNP – n-terminal prohormone of brain natriuretic peptide, EDVi/ESVi – end-diastolic/-systolic volume index, EF – ejection fraction, GLS – global longitudinal strain, MPA_Puls_ – Main pulmonary artery pulsatility, MPA_Cap_ – Main pulmonary artery capacitance, ECV – Extra cellular volume, LV/RV – Left/Right ventricular

Supplemental Table 2: Characteristics of patients with low and high main pulmonary artery capacitance

| Parameter | MPA_Cap_ ≤0.22%/ml (n=44) | MPA_Cap_ >0.22%/ml (n=19) | p-value |
| --- | --- | --- | --- |
| Baseline characteristics | | | |
| Age (years) | 69.0 (66.0;75.0) | 69.0 (66.5;72.3) | 0.133 |
| BMI (kg/m^2^) | 28.1 (26.2;32.4) | 28.0 (25.2;31.2) | 0.339 |
| ntProBNP (ng/l) | 150.1 (70.3;281.0) | 99.2 (47.5;152.4) | 0.004 |
| HFpEF (n,%) | 23 (52) | 7 (37) | 0.260 |
| Male sex (n,%) | 18 (41) | 6 (32) | 0.484 |
| NYHA II/III (n,%) | 31/13 (71/29) | 13/6 (68/32) | 0.872 |
| Diabetes (n,%) | 7 (16) | 2 (11) | 0.575 |
| AHT (n,%) | 37 (84) | 14 (74) | 0.334 |
| AFIB (n,%) | 18 (41) | 2 (11) | 0.017 |
| DLP (n,%) | 25 (57) | 14 (74) | 0.206 |
| Smoking (n,%) | 7 (16) | 2 (11) | 0.575 |
| HFA PEFF Score | 4 (4;6) | 3 (2;5) | 0.006 |
| Echocardiography | | | |
| E/e’ Rest | 10.9 (9.1;12.9) | 8.7 (7.7;9.8) | 0.021 |
| E/e’ Stress | 13.2 (10.4;16.7) | 11.5 (10.1;13.6) | 0.034 |
| LAVI (ml/m^2^) | 46.8 (37.2;58.2) | 40.1 (30.2;47.5) | 0.094 |
| TAPSE (mm) | 23.7 (21.4;27.1) | 32.2 (19.5;26.2) | 0.835 |
| PAPsys (mmHg) | 24.0 (21.2;31.2) | 24.2 (21.0;30.5) | 0.051 |
| Right heart catheterization | | | |
| PCWP rest (mmHg) | 11.0 (9.5;17.0) | 8.0 (5.3;13.8) | 0.029 |
| PCWP rtress (mmHg) | 26.0 (22.0;29.5) | 19.5 (13.0;22.0) | 0.016 |
| mPAP rest (mmHg) | 200;17.0;25.5) | 17.0;15.3;22.5) | 0.062 |
| mPAP stress (mmHg) | 35.5;42.0;48.0) | 36.0 (28.8;40.5) | 0.042 |
| PVR rest (Wood) | 1.6 (1.0;2.2) | 1.7 (1.4;2.1) | 0.589 |
| PVR stress (Wood) | 1.5 (1.1;2.3) | 1.5 (1.3;2.2) | 0.030 |
| Cardiovascular Magnetic Resonance Imaging | | | |
| LVMI (g/m^2^) | 60.0 (52.0;71.7) | 56.8 (47.4;64.4) | 0.919 |
| LVEDVi (ml/m^2^) | 71.0 (61.8;76.2) | 75.1 (55.6;82.3) | 0.726 |
| LVESVi (ml/m^2^) | 20.3 (16.2;25.8) | 20.9 (13.2;27.3) | 0.287 |
| LVEF (%) | 68.6 (65.0;75.8) | 68.6 (65.0;75.8) | 0.307 |
| LV GLS (%) | -20.6 (-23.1;-18.9) | -20.6 (-22.7;-18.9) | 0.880 |
| RVEDVi (ml/m^2^) | 68.9 (59.2;76.5) | 67.5 (49.7;83.1) | 0.521 |
| RVESVi (ml/m^2^) | 23.4 (18.4;27.9) | 19.5 (16.3;32.5) | 0.435 |
| RV GLS | -23.4 (28.2;-21.6) | -22.3 (-25.3;-20.1) | 0.265 |
| RVEF (%) | 65.5 (62.2;68.9) | 68.9 (59.1;71.5) | 0.850 |
| T1 (ms) | 1198 (1184;1223) | 1198(1185;1227) | 0.297 |
| ECV (%) | 25.7 (24.0;27.9) | 25.5 (23.9;27.7) | 0.180 |

Values are provided in frequencies with corresponding percentages for categorical variables or as a median with corresponding inter-quartile range for continuous variables. BMI – Body mass index, HFpEF – heart failure with preserved ejection fraction, TAPSE – Tricuspid annular plane systolic excursion, E – passive mitral inflow, e’ – septal and lateral mitral annulus velocity, PCWP – pulmonary capillary wedge pressure, mPAP – mean pulmonary artery pressure, , PVR – pulmonary vascular resistance, PAPsys – pulmonary artery systolic pressure, LVMI – left ventricular mass index, LAVI – left atrial volume index, ntPro-BNP – n-terminal prohormone of brain natriuretic peptide, EDVi/ESVi – end-diastolic/-systolic volume index, EF – ejection fraction, GLS – global longitudinal strain, MPA_Puls_ – Main pulmonary artery pulsatility, MPA_Cap_ – Main pulmonary artery capacitance, ECV – Extra cellular volume, LV/RV – Left/Right ventricular
